# Apolipoprotein A5 reduces clearance of VLDL by altering apolipoprotein E content

**DOI:** 10.1016/j.jlr.2025.100917

**Published:** 2025-10-01

**Authors:** Pheruza Tarapore, Debi Swertfeger, Jamie Morris, Yi He, Snigdha Sarkar, John T. Melchior, Amy S. Shah, Min Liu, W. Sean Davidson

**Affiliations:** 1 Department of Pathology and Laboratory Medicine, University of Cincinnati, Cincinnati, OH, USA; 2 Department of Pediatrics, Cincinnati Children’s Hospital Medical Center and the University of Cincinnati, Cincinnati, OH, USA; 3 Department of Medicine, University of Washington School of Medicine, Seattle, WA, USA; 4 Biological Sciences Division, Earth and Biological Sciences Directorate, Pacific Northwest National Laboratory, Richland, WA, USA

**Keywords:** apolipoprotein, apolipoprotein A-V, apolipoprotein E, lipolysis, LPL, VLDL, triglyceride-rich, MS, structure

## Abstract

Apolipoprotein A-V (APOA5) is a critical regulator of circulating triglyceride (TG) levels. Its deletion leads to elevated plasma TG concentrations by altering the metabolism of VLDL particles in vivo. One way APOA5 exerts its effects is through the modulation of LPL activity, specifically by disrupting inhibitory interactions between LPL and angiopoietin-like proteins (ANGPTLs). However, the impact of APOA5 on VLDL composition and its potential to alter VLDL metabolism in other ways remains poorly understood. To address this, we investigated the influence of APOA5 on the VLDL proteome, LPL activation, and hepatic remnant uptake. Using VLDL from *Apoa5* KO and WT mice, we found no evidence that APOA5 directly enhances LPL activity in purified or plasma systems. However, VLDL from *Apoa5* KO mice was cleared significantly more slowly by cultured hepatocytes. VLDL proteomics experiments from two independent laboratories identified altered contents of 23 proteins involved in lipoprotein metabolism, inflammation, and immune response in *Apoa5* KO VLDL, including reductions in APOE and serum amyloid A1. Remarkably, reintroduction of recombinant mouse APOA5 to the KO plasma partially restored the WT VLDL proteome, including APOE, and normalized VLDL uptake by hepatocytes without altering LPL lipolysis. These findings reveal that APOA5 influences hepatic clearance of VLDL remnants by modulating particle composition, particularly APOE content. This study expands the functional scope of APOA5 in TG metabolism and underscores its role in VLDL remodeling and remnant clearance, offering new insights with implications for understanding hypertriglyceridemia and its roles in inflammation and immune response.

Apolipoprotein A-V (APOA5) is a 39 kDa protein that plays a major role in regulating circulating triglycerides (TGs) in humans and mice (1, 2). In mice, *Apoa5* knockdown results in a 2-fold increase in plasma TG levels, whereas its overexpression leads to an approximately 70% reduction of TGs (3). APOA5 polymorphisms in humans contribute to interindividual variation in circulating TG in numerous populations (4, 5, 6). As part of the gene expression cassette, which includes APOA1, APOA4, and APOC3, most APOA5s are expressed in the liver, where they may play a role in TG conservation, particularly in instances of liver damage or regeneration (7). Despite its importance, APOA5 is present in the circulation at relatively low concentrations, ranging from 20 to 500 ng/ml (8), which is about four orders of magnitude lower than APOA1, the primary protein on HDL, and three orders lower than APOB, the primary protein on LDL and VLDL. This translates to roughly one APOA5 molecule per ∼25 VLDL particles. Moreover, circulating APOA5 is distributed roughly equally between VLDL and HDL and is also found in chylomicrons when present (8, 9).

Low plasma concentrations of APOA5 on TG-rich lipoprotein (TGRL) have made it challenging to decipher the mechanistic basis of its influence on TGRL secretion, processing, and uptake. While early findings were somewhat inconsistent (10), most now agree that APOA5 has little effect on TGRL secretion from the liver (11, 12). Instead, it likely regulates TG levels postsecretion through at least two effects on LPL (10, 12, 13), the enzyme responsible for hydrolyzing TG to release free fatty acids for peripheral uptake. The first mechanism involves modulation of LPL catalytic activity. While earlier work suggested that APOA5 might directly bind to and stimulate LPL (12, 13), more recent evidence indicates that APOA5 instead competes with LPL for binding to the inhibitory angiopoietin-like proteins (ANGPTL3/8 complex), effectively acting as a derepressor (14). This is mediated via the C-terminal 40 residues of APOA5 (15). The second mechanism involves regulation of LPL abundance and localization within the capillary bed. Mice lacking APOA5 have reduced levels of LPL in capillaries and tissues that utilize fatty acids, likely due to the same APOA5 interaction with the ANGPTL3/8 complex (16). When active, ANGPTL3/8 promotes the dissociation of LPL from its binding sites on endothelial cells, but APOA5 binding prevents this, helping retain LPL in the capillary lumen. Additionally, APOA5 interacts with glycosylphosphatidylinositol HDL binding protein 1 (GPIHBP1), an endothelial cell protein that transports LPL from the subendothelial space to the capillary surface (17). Through these mechanisms, APOA5 plays key roles in both the activity and localization of LPL.

Aside from effects on LPL, there are suggestions that APOA5 can also facilitate the clearance of remnant particles in the liver. Grosskopf *et al.* (11) showed that VLDL particles isolated from *Apoa5* KO mice were cleared from the plasma at a slower rate than WT VLDL, even when exposed to liver cells with normal remnant receptor activity. As these particles tended to be larger and TG enriched, they suggested that proteomic differences were responsible for poorer binding to remnant receptors and thus delayed clearance. Indeed, their SDS gel analyses suggested that VLDL from *Apoa5* KO mice may be depleted in APOE and enriched in one or more of the APOCs. The authors speculated that the lack of APOA5 causes an alteration of the lipoprotein proteome in the hepatic sinusoid, resulting in delayed receptor uptake. Unfortunately, the impacts of *Apoa5* KO on VLDL proteomic compositions were never followed up in detail. Here, we carefully characterized the proteome of VLDL in the presence or the absence of APOA5 and, taking advantage of our ability to produce recombinant mouse APOA5 (r-APOA5), evaluated the effects of different levels of APOA5 on the *Apoa5* KO mouse VLDL proteome by tracking the ability of the particles to be hydrolyzed by LPL or taken up in liver cells. We found that APOA5, despite its low plasma levels, impacts the VLDL proteome in surprising ways, including altering the amount of APOE, which can affect cellular uptake and remnant particle clearance.

## Materials and methods

### Animals and plasma collection

Animals were maintained in a pathogen-free facility, and all experimental procedures were performed with protocols approved by the University of Cincinnati Institutional Animal Use and Care Committee and in accordance with the National Institutes of Health Guidelines for the Care and Use of Animals. All mouse studies reported here involved mice on normal chow diets. All mice were maintained on a 12-h light and dark cycle under controlled environmental conditions with free access to food and water. *Apoa5* KO mice on a C57BL/6 background were obtained from Dr Patrick Tso and Min Liu at the University of Cincinnati (1). To study the effect of APOA5 on TG metabolism, 21-week-old male mice were fasted for 9 h prior to receiving an intravenous injection of either lipidated mouse APOA5 (530 ng/g body weight or 30 ng/g body weight) or vehicle as a control. Blood was collected before and at 1, 2, 3, and 4 h after intravenous injection via the tail vein, into heparinized tubes. Samples were centrifuged at 3,000 *g* for 10 min to remove cellular components, and plasma was stored at 4°C before TG measurements were performed. For all other mouse experiments, mice were fasted for at least 5 h before blood collection.

### Lipoprotein, protein, and lipid measurements

Mouse APOA5 levels were determined using the APOA5 ELISA Kit (Mouse) from Aviva Systems Biology (San Diego, CA). Mouse APOB levels were determined using the Mouse ApoB ELISA Kit (Abcam, Boston, MA). Protein levels were determined using the Markwell modification of the Lowry protein assay (18). TG and total cholesterol levels were measured with enzymatic kits from Pointe Scientific. Phosphatidylcholine levels and NEFA levels were measured using the commercially available kits (FUJIFILM Wako Pure Chemical Corporation, Japan).

### Preparation of lipidated mouse ApoA5

r-APOA5 was prepared as described for human APOA5 (19). Briefly, mouse APOA5 protein was expressed in ClearColi BL21 (DE3) cells (Research Corporation Technologies, Inc) in 200 ml LB broth with ampicillin. At an absorbance of 0.8 at 600 nm, cultures were induced with 0.4 mM isopropyl β-d-1-thiogalactopyranoside for ∼4 h. Cells were pelleted by centrifugation (9,800*g*), lysed, and purified using nickel affinity chromatography as described (19). Lipidated APOA5 particles (APOA5/POPC) were generated with a modified sodium cholate dialysis method as previously described (20) using deoxycholic acid sodium salt (Fisher Scientific). r-APOA5 protein in ammonium bicarbonate and 3 mM DTT was incubated with phospholipid, POPC (Avanti Polar Lipids), at a molar ratio of 125:1 POPC:protein at a 1 mg/ml protein concentration to generate APOA5/POPC particles. They were then dialyzed into ammonium bicarbonate in nonreducing conditions. The particle sizes and homogeneity were confirmed by 4–16% nondenaturing gradient gel electrophoresis (Invitrogen) with Coomassie blue staining. Protein concentrations of samples containing 3 mM DTT were determined by the Bradford protein assay, and particle concentrations were determined by the Markwell-Lowry assay.

### Generating stable LPL-expressing cells and measurements of VLDL TG lipolysis in mice

Human embryonic kidney 293 cells were maintained in DMEM with 10% FBS and penicillin-streptomycin. Cells were stably transfected with human LPL. pSS1 (hLPL-FLAG) was a gift from Brandon Davies (Addgene plasmid #102447; http://n2t.net/addgene:102447; Research Resource Identifier: Addgene_102447) (21), and the cells expressing LPL were selected using blasticidin. The LPL-positive colonies were identified by Western blot with rabbit anti-FLAG antibody (#F3165; MilliporeSigma). Since LMF1 (lipase maturation factor 1) can influence LPL’s post-translational maturation and activity, the LPL-expressing cells were further stably transfected with pCMV-LMF1 V5-Myc-tag plasmid (kind gift of Dr Miklos Peterfy) (22). Cells were selected using G418, and colonies were identified by Western blot using rabbit anti-myc antibody (#2278, 1:1,000 dilution; Cell Signaling Technology, Inc). LPL was released from the cell surface using heparin sodium derived from porcine mucosa (20 units/ml, 1 h at 37 C; Sigma), and the media containing LPL were collected and concentrated (Amicon, 30 kDa) while washing out the heparin. Lipolysis reactions included either plasma (5 μg TG/10 μl reaction) or VLDL (10 μg TG/10 μl reaction). VLDL or plasma TG lipolysis was assessed by measuring released free fatty acids using an NEFA assay (FujiFilm Wako Pure Chemical Corporation, Japan).

### Immunoblotting

Equal volumes of plasma fractions were loaded onto SDS-PAGE gels. VLDL samples were loaded at equal ApoB concentrations as determined by the ELISA assay above. Gels were transferred to PVDF Immobilon-P transfer membrane using the Transblot SD Semidry transfer cell (Bio-Rad). Membranes were blocked using 5% nonfat dried milk in 0.5% Tween-20 in TBS, pH 7.5, and incubated overnight at 4°C with the appropriate primary antibody. The primary antibodies used were rabbit-anti-mouse ApoB100 (GTX135994, 1:1,000 dilution; GeneTex), rabbit-anti-mouse Apoa5 (#OACD01724, 1:1,000 dilution; Aviva Systems Biology), rabbit-anti-mouse Apoa1 (#PA5-29557, 1:1,000 dilution; Thermo Fisher), rabbit-anti-mouse Apoe (#68587, 1:2000 dilution; Cell Signaling Technology), goat serum amyloid A1/A2 (SAA1/SAA2) (#AF2948, 1:500 dilution; R&D Systems), and rabbit-anti-mouse Apod (#Ab187513, 1:500 dilution; Abcam). The secondary antibody used was HRP-AffiniPure donkey anti-rabbit-IgG (#711-035-152; Jackson ImmunoResearch Laboratories, Inc) at a 1:2,000 dilution for 1 h at room temperature.

### Isolation of VLDL by ultracentrifugation for MS

To isolate 300 μg of VLDL protein, either 2.5 ml (*Apoa5* KO mice) or 4.7 ml (WT mice) plasma was mixed with a density of 1.0063 g/ml saline solution, placed in a Ti 50.4 rotor, and centrifuged at 40,000 *g* for 17 h at 5°C (n = 4/group). Each sample consisted of pooled plasma from four fasted mice. For all applications, plasma was age-matched within and between groups such that each group had mice between 20 and 32 weeks of age.

### Sample preparation for MS

VLDL samples undergoing MS analysis were dialyzed into 50 mM ammonium bicarbonate (pH 8.1) with 5 mM DTT, and protein concentration was determined by the Bradford assay. Preparation methods were modified from previously described methods (23, 24, 25). Briefly, VLDL samples were distributed into 100 μl aliquots, adjusted to 5% sodium deoxycholate (SDC) and 10 mM DTT for 30 min at 60°C. Iodoacetamide was added to the samples to a final concentration of 40 mM for 60 min at room temperature, protected from light. The samples were then diluted to an SDC concentration of 1% and digested overnight with trypsin. The next day, following an additional 1 h tryptic digestion, formic acid was added to a final concentration of 2% formic acid to precipitate lipids and SDC. The samples were then centrifuged at 15,000 *g* at 4°C for 15 min. The supernatants were carefully removed and lyophilized to dryness by SpeedVac. Prior to MS analysis, peptides were desalted using ultramicrospin C18 columns (Nest Group, Ipswich, MA). Briefly, peptide mixtures were loaded onto pre-equilibrated columns, which were washed four times with 5% acetonitrile, and desalted samples were eluted with 80% acetonitrile. Eluted peptides were concentrated using a SpeedVac, and final concentrations were determined using the Pierce BCA protein assay kit (catalog no.: 23225). Samples were diluted to 0.1 mg/ml and stored at −20°C until MS analysis.

### Mass spectrometry

#### Site A, University of Washington

Capillary LC-ESI-MS/MS was performed using an IntegraFrit capillary C18 trapping column (Waters XBridge BEH C18, 5 μm, 0.1 × 40 mm), a capillary analytical C18 column packed in-house (Waters XBridge BEH C18, 5 μm, 0.075 × 250 mm), and an Orbitrap Fusion Lumos Tribrid mass spectrometer (Thermo Electron, Bremen, Germany). The column was kept at 37°C, and nanoACQUITY UPLC (Waters, Milford, MA) was used for the separation, with a linear gradient of 0.1% formic acid in water (solvent A) and 0.1% formic acid in 80% acetonitrile (solvent B). In each experiment, the digested sample was desalted on a trapping column for 10 min at 2 μl/min in 98% solvent A. Effluent from the trapping column was directed to the analytical column at a flow rate of 0.3 μl/min and separated using a gradient as follows: 8–25% solvent B in 40 min and 25–45% solvent B in 20 min. The column was subsequently washed for 10 min at 100% B and re-equilibrated at 98% A for 15 min. Eluting peptides were electrosprayed into an Orbitrap Fusion Lumos Tribrid mass spectrometer operating in the data-dependent mode to acquire a full MS scan (350–1,600 *m/z*) and then subsequent MS/MS scans of precursor ions by their intensity. MS1 scans were acquired in the Orbitrap, and the resolution was set at 120,000. MS/MS scans were acquired in the Orbitrap with a resolution of 30,000. Isolation width was set at 1.6 *m/z*, the automatic gain control target at 5.0 × 10^4^, and the maximum injection time at 50 ms. Higher-energy collisional dissociation was performed at 30% normalized collision energy.

#### Site B, Pacific Northwest National Laboratory

Peptide mixtures (0.1 mg/ml) were analyzed on an Orbitrap Q Exactive Mass Spectrometer (MS) (ThermoFisher Scientific, San Jose, CA) coupled to an in-house built ultra-HPLC using an autosampler (PAL) Valco injection pods (Valco) and Dionex Ultimate 3000 pumps (Thermo Scientific, San Jose, CA). Samples were first loaded onto a solid-phase extraction column packed in-house by slurry packing 5-μm Jupiter C18 (Phenomenex, Torrance, CA) into 5 cm × 360 μm o.d. × 150 μm i.d fused silica at a flow rate of 5 μl/min. Mobile phases consisted of 0.1% formic acid in water (A) and 0.1% formic acid in acetonitrile (B) (Optima LC-MS grade; Fisher Scientific, Saint Louis, MO) operated at a flow rate of 200 nl/min. After solid-phase extraction desalting, the 5 μl injection volume was focused onto a reversed-phase column packed in-house (1.7-μm Waters BEH C18; Waters Corporation, Milford, MA) using 30 cm × 360 μm o.d. × 75 μm i.d fused silica with a fully integrated tip (CoAnn Technologies, Richland, WA) heated to 50°C. Peptides were eluted over 120 min with a gradient profile as follows (min: %B); 0:5.0, 2:8.0, 20:12.0, 75:25, 97:60, and 100:75, with a 15 min start delay. The gradient was followed by a wash and equilibration. Spectra were acquired for 300–1,800 *m/z* with a normalized collision energy set to 30%.

### Analyses of MS data

Quantitation was performed using MaxQuant (version 1.6.8) with unmodified .raw files transferred from sites A and B, allowing match between runs, three peptides per protein, and a 5% false discovery rate (26). Peptide spectral data were searched against the UniProtKB/Swiss-Prot Protein knowledgebase (mouse C57Bl6 08/05/2021; 17,077 entries) using MaxQuant. Data were constrained to tryptic digestion with a maximum of three missed cleavages. Carbamidomethylation was set as a fixed modification, Met oxidation as a variable modification, and acetylation of the N terminus was allowed. Peptide and MS/MS mass tolerance was ±0.15 Da. Proteins and peptides were constrained to >99.9% and 95% identification probability, respectively. In addition, proteins were only accepted if they contained a minimum of three unique peptides. Statistical analyses were performed using Perseus, version 2.0.10.0, after normalization to the apoB label-free quantitation (LFQ) counts found within each respective sample to determine per-particle changes in LFQ counts. The protein identifications classified as only identified by site, contaminants, and reverse were excluded from analysis. The expression values were Log2 transformed to facilitate the visualization of protein abundance fold differences. Since there were eight total samples, the level of stringency for our analysis was defined as the minimum number of valid values in at least seven groups in total. Data were filtered based on the total coverage of peptides on the protein >10%. Significance was calculated by adjusting for multiplicity tests (Benjamini-Hochberg) using the permutation-based false discovery rate cutoff of 13%. The MS proteomics data obtained from both sites A and B have been deposited to the ProteomeXchange Consortium via the PRIDE (27) partner repository with the dataset identifier PXD065274.

### Measurement of VLDL uptake in cultured liver cells

Plasma from WT and Apoa5 KO mice was incubated with 80 μg/ml of 1,1′-dioctadecyl-3,3,3′,3′-tetramethylindocarbocyanine perchlorate (DiI) overnight at 37°C. The next day, *Apoa5* KO plasma was divided into three parts and incubated for 1 h at 37°C with either 500 ng/ml, 2.5 μg/ml, or 0 ng/ml mouse APOA5. Samples were immediately size separated using a Superose 6 Increase chromatography column, and the fractions corresponding to VLDL were pooled. The TG and ApoB levels were determined. Mouse Alpha Mouse Liver 12 hepatocyte cell line was obtained from ATCC (Manassas, VA) and maintained in DMEM with 10% FBS and penicillin-streptomycin. Cells were seeded into 48-well plates at 25,000 cells/well and serum starved for 2 h before the addition of 0–20 μg/ml of DiI-VLDL for 1 h at 37°C. Cells were washed twice with PBS. Cells were lysed using 1% Triton X-100 lysis buffer with shaking at 42°C for 2 h, and fluorescence was measured.

### Statistical analysis

Statistical analyses were performed and graphics generated with SigmaPlot, version 11.2 (Systat Software, Inc). For most analyses, differences from the negative control were determined by a repeated-measures mixed model ANOVA with a Bonferroni post-test. Some experiments used a Student’s *t*-test to calculate significance, as described in the figure legends.

## Results

### Characterization of the mouse model and the effectiveness of r-APOA5

**Fig. 1:**
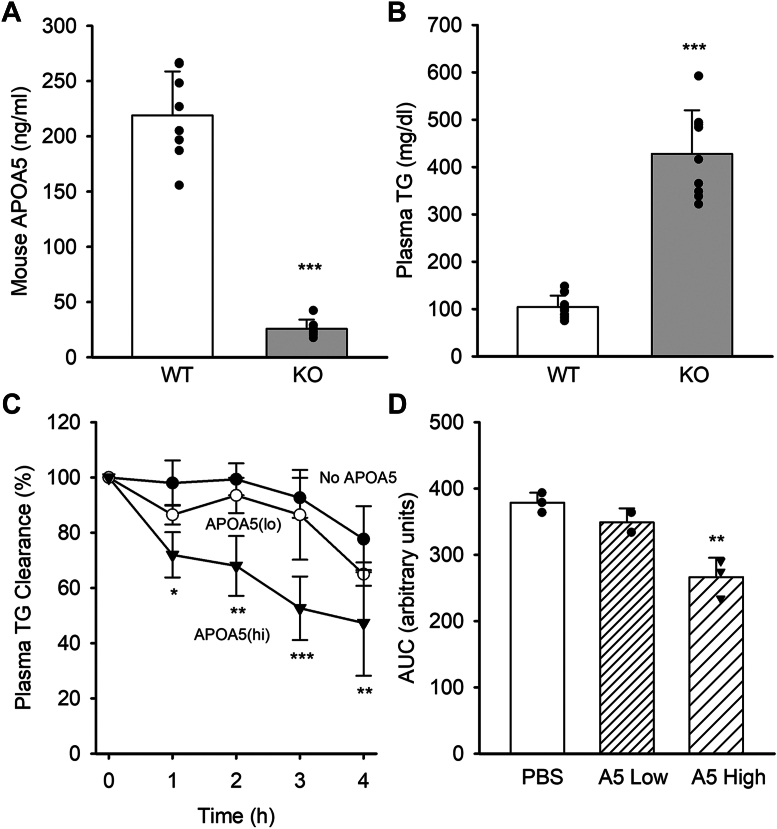
Characterization of *Apoa5* KO mouse model and effectiveness of r-APOA5. A: Mouse APOA5 protein levels in plasma were determined by APOA5 ELISA (n = 7 mice per group). ∗∗∗ indicates significant difference from WT at *P* < 0.0001 by Student’s *t*-test. B: Plasma TG concentrations were determined by colorimetric assay (n = 9 mice per group). ∗∗∗ indicates significant difference from WT at *P* < 0.0001 by Student’s *t*-test. C: Intravenous injection of r-APOA5/POPC complexes into *Apoa5* KO mice. *Apoa5* KO mice were fasted for 9 h before intravenous injections of PBS control (black circles), low-dose APOA5/POPC (30 ng/g body weight, open circles), or high-dose APOA5/POPC (530 ng/g body weight, filled triangles). Plasma samples were collected via the tail vein at the indicated time points and analyzed for TG (n = 3 per group). Differences from vehicle control were determined by a repeated-measures mixed model ANOVA with Bonferroni post-test *P* value ≤0.05∗, ≤0.01∗∗, and ≤0.005∗∗∗. Values are shown as percentages with T = 0 h set to 100% (starting values were PBS control, 303 ± 29 μg/ml TG; low dose A5, 260 ± 11 μg/ml TG; high dose A5, 273 ± 8 μg/ml TG). D: The area under each curve was determined by summing the data for time points 1–4 in C. ∗∗ shows a difference from control by one-way ANOVA at *P* < 0.01. All values are expressed as means, and the error bars show 1 sample SD.

Minimal mouse APOA5 was detectable in the *Apoa5* KO mice compared with about 220 ng/ml in age-matched WT mice (Fig. 1A). In agreement with previous work (28), *Apoa5* KO mice exhibited approximately 4-fold increased circulating TG versus WT mice (Fig. 1B). Size-exclusion chromatography analysis of both WT and *Apoa5* KO plasma showed no significant difference in VLDL particle size under fasted conditions (Supplemental Fig. S1), despite its high abundance in the KO mice. In previous studies, we have shown that human APOA5 produced in our *Escherichia coli* expression system could effectively normalize the clearance rate of plasma TG after a fatty gavage in *Apoa5* KO mice (19). Since mice were used as the experimental model for the current studies, we produced mouse APOA5 in the same system (see Methods section). In general, we observed a similar level of expression and yield with the mouse sequence versus human and overall similar stabilities at physiological pH, as long as bicarbonate buffers were used (19). Figure 1C, D show that injection of r-APOA5 complexed to POPC lowered TG levels in mouse plasma over 4 h, particularly at the higher dose of 530 ng/g body weight. This indicates that our r-APOA5 exhibits the expected biological activity in mice.

**Fig. 2:**
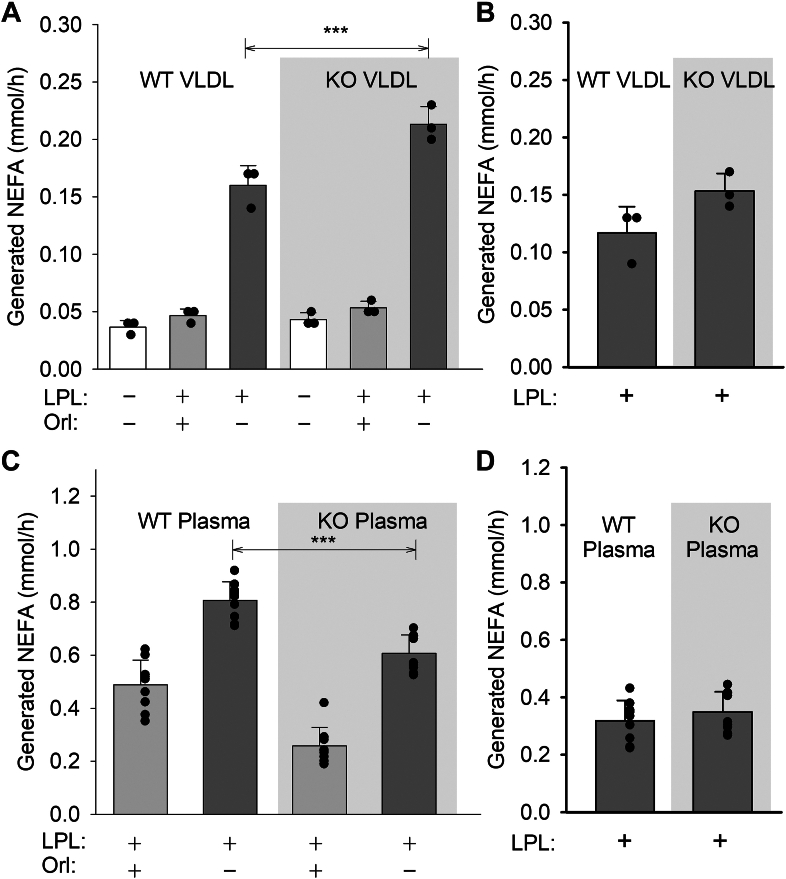
TG lipolysis in VLDL and plasma of WT and *Apoa5* KO mice. A: VLDL was isolated from WT and *Apoa5* KO mice by ultracentrifugation and evaluated (at equal apoB concentrations) as substrates for exogenous LPL in the presence (negative control) and absence of orlistat (Orl), a pan lipase inhibitor. A colorimetric assay for NEFAs was used. Differences between WT and KO were determined by one-way ANOVA (Bonferroni post-test). ∗∗∗ reflects *P* ≤ 0.005. B: The efficiency of lipolysis in VLDL after subtracting the corresponding negative control. C: Plasma from both genotypes was examined for efficiency of lipolysis by exogenous LPL and (D) was compared after normalizing for the appropriate negative control. Experiments in each panel were performed at least twice with representative data shown. Significance determined by a Student's *t*-test. *P* ≤ 0.01∗∗ and ≤0.005∗∗∗. All values are expressed as mean ± SD.

We next sought to confirm the observation of Grosskopf *et al.* that VLDL particles isolated from WT are more effective substrates for exogenous LPL versus VLDL isolated from *Apo*a*5* KO mice (11). Figure 2A shows that the NEFA generated by LPL activity was minimal in the absence of added LPL or in the presence of the pan lipase inhibitor orlistat in both genotypes. In the presence of active LPL, NEFA levels increased about 4-fold for WT and about 5-fold for the *Apoa5* KO. In this simple system, this confirms that APOA5 on VLDL does not directly stimulate LPL activity. In fact, in contrast to Grosskopf’s study, we were surprised to note that the VLDL particles from the KO mice, whether compared at equal TG or APOB concentrations, were slightly more effective LPL substrates than WT VLDL (Fig. 2B). We next looked at the exogenous LPL activity in the more complex environment of plasma from WT or *Apoa5* KO mice (Fig. 2C). In this case, WT VLDL and KO VLDL were equally effective as LPL substrates, as shown in Figure 2D, despite the lower basal NEFA levels in KO plasma (Fig. 2C). Overall, our data demonstrate that APOA5 does not directly stimulate LPL and support the proposal (14) that APOA5 acts through additional factors present in plasma (such as ANGPTL 3/8) to enhance VLDL lipolysis.

### VLDL from Apoa5 KO mice is cleared more slowly than WT in cultured mouse hepatocytes

**Fig. 3:**
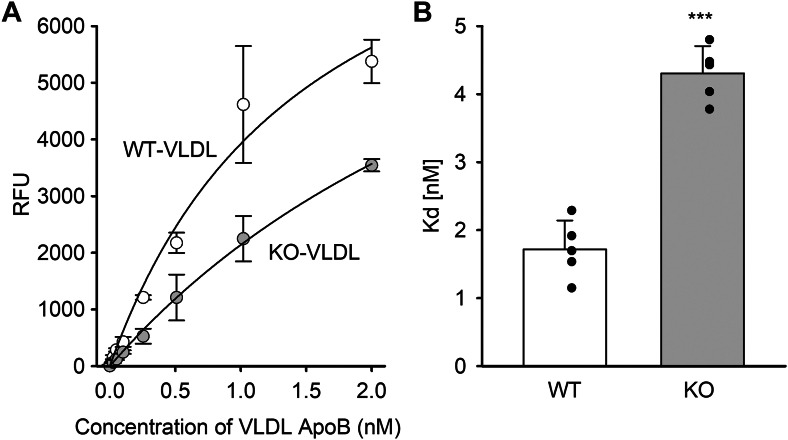
Uptake of VLDL from WT or *Apoa5* KO into cultured hepatocytes. A: VLDL isolated by size-exclusion chromatography from WT and *Apoa5* KO mice or by ultracentrifugation was labeled with DiI and evaluated for binding to AML12 cells (a mouse liver hepatocyte line) at increasing concentrations of APOB. B: Apparent *K*_*d*_s were determined by fitting the data in A to a single-site binding model (GraphPad Prism). The figure represents an average of two experiments performed in duplicate. The values were normalized to cell protein, the intensity of fluorescence labeling and APOB levels between the groups. Significance was calculated using Student’s *t*-test as follows: ∗∗∗*P* < 0.0001. All values are expressed as mean ± SD.

Using cultured Alpha Mouse Liver 12 cells (a mouse liver hepatocyte line), we evaluated the ability of ultracentrifugally isolated VLDL from WT or *Apoa5* KO mice to bind to remnant receptors and be cleared from the medium. Figure 3A shows that DiI-labeled VLDL from WT mice effectively bound to the cells with an apparent *K*_*d*_ of clearance of about 1.6 nM (Fig. 3B). However, VLDL from *Apoa5* KO VLDL, compared at the same level of apoB (i.e., same particle number), was cleared more slowly with an apparent *K*_*d*_ of clearance of >4 nM.

### Lack of APOA5 results in significant proteomic alterations in mouse VLDL

**Fig. 4:**
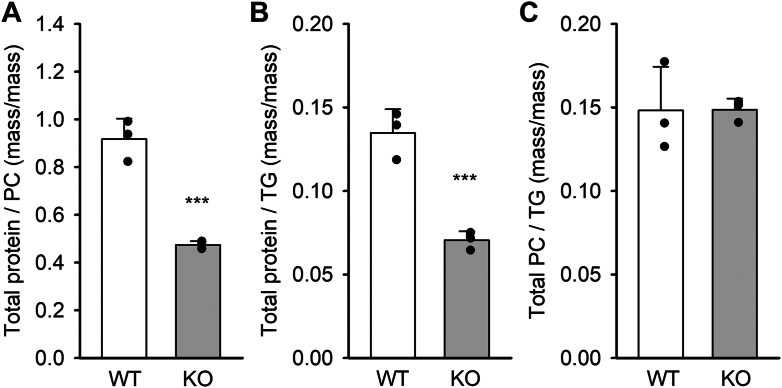
Protein and lipid content of VLDL from WT and *Apoa5* KO mice. A: VLDL isolated by ultracentrifugation was matched for APOB concentrations using an ELISA assay and then assayed by Markwell-Lowry assay for protein and a colorimetric choline assay for phosphatidylcholine. B and C: TG levels were measured using a colorimetric kit. Significance was determined using Student’s *t*-test as follows: ∗∗∗*P* < 0.0001. All values are expressed as mean ± SD.

The difference in particle uptake between VLDL particles isolated from WT versus *Apoa5* KO mice strongly suggested that compositional changes in the particle proteome may be responsible. We analyzed ultracentrifugally isolated VLDL from both genotypes with respect to bulk protein and lipid contents. Figure 4 shows that *Apoa5* KO VLDL was depleted in total protein with respect to its phosphatidylcholine content (Fig. 4A) and its TG content (Fig. 4B). However, the ratio of PC to TG was unaltered between the groups. Thus, the lack of APOA5 results in an overall loss of protein in VLDL compared with WT.

**Fig. 5:**
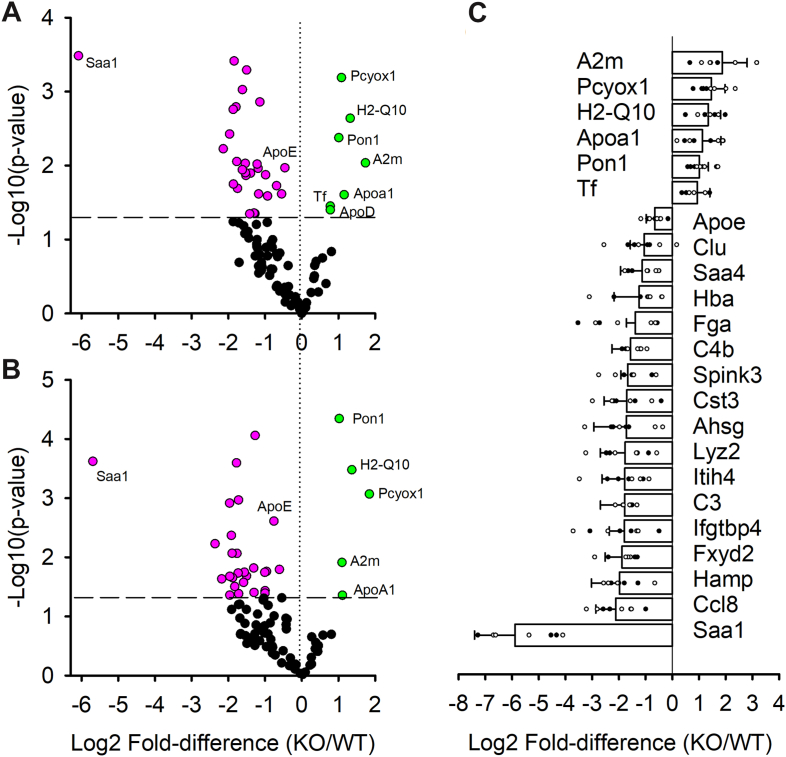
Proteomic analysis of isolated VLDL from WT and *Apoa5* KO mice. Four pooled plasma samples were prepared, each resulting from four age-matched and fasted mice. VLDL was isolated by ultracentrifugation, and 100 μg of VLDL (determined by modified Markwell-Lowry assay) was delipidated for electrospray MS in two independent laboratories. Peptide spectral data were searched against the UniProtKB/Swiss-Prot Protein database (see Methods section). LFQ intensities were normalized to APOB counts. A: Volcano plot for samples processed at the University of Washington. Each dot shows one identified protein. Proteins in black showed no statistical difference in abundance at a *q* value of 0.13. Proteins in magenta were enriched in the WT sample, whereas those in green were more prevalent in *Apoa5* KO VLDL. The dashed line shows the *P* value cutoff for significance. B: Volcano plot for samples processed at Pacific Northwest National Laboratories set up the same way as A. C: Difference plot of proteins determined to be statistically different in both analyses (n = 8). Values are expressed as mean ± SD.

To determine how the chronic absence of APOA5 affected individual proteins in VLDL, we performed proteomic analyses using MS. To maximize rigor, we performed this analysis at two independent laboratories with independent samples, one at the University of Washington (site A) and the other at Pacific Northwest National Laboratories (site B, see Methods section). The samples were prepared at equal TG amounts. The LFQ intensities were adjusted for equal APOB to compare per-particle counts and to compensate for minor differences in sample introduction into the instruments. Figure 5A, B shows volcano plots for both analyses. Site A generated quantifiable data on 100 proteins, whereas site B tracked 90 proteins. Both methods found that *Apoa5* KO VLDL was statistically enriched in alpha 2-macroglobulin (A2M), PCYOX11, H2-Q10, APOA1, PON1, and transferrin versus WT (green points). Site A additionally found that APOD was enriched, but this protein was not quantified in the site B workflow. By contrast, 17 proteins were depleted (magenta points) in *Apoa5* KO VLDL versus WT in both analyses (listed in Fig. 5C). These include proteins involved in lipoprotein metabolism like APOE, clusterin (APOJ), and SAA4. Strikingly, the protein with the highest fold enrichment in WT versus *Apoa5* KO VLDL was SAA1, an acute phase response protein typically associated with infection, inflammation, or injury (29). However, many of the altered proteins play roles in other processes. A Gene Ontology search indicated that most are associated with the complement and coagulation cascades. Others participate in antigen processing and presentation as well as other pathogen infections (Supplemental Fig. S2).

**Fig. 6:**
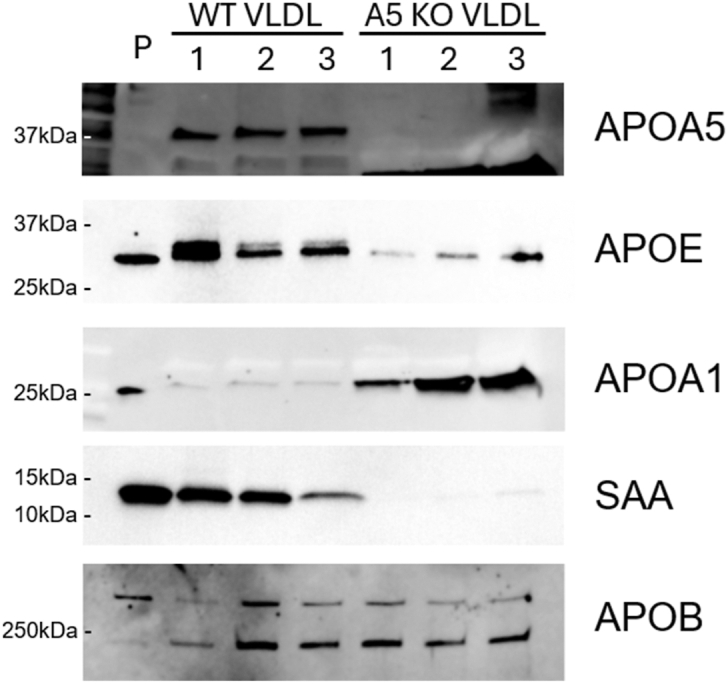
Verification of key protein abundance differences assessed by Western blot. Ultracentrifugally isolated VLDL from WT or *Apoa5* KO mice was prepared, assayed for APOB content by ELISA, separated by SDS-PAGE, and probed with antibodies for APOA5, APOE, SAA (shown to be reduced in the KO by MS in Fig. 5), and APOA1 (increased in KO VLDL). Diluted unfractionated WT mouse plasma (P) was used to show the correct size of each protein. Each numeral represents a single mouse.

For added confidence, we verified the levels of select proteins independently by Western blot. Figure 6 shows that APOA5 was not detected in the KO mice as expected. As indicated in both MS analyses, APOA1 levels were clearly enriched in the VLDL particles from *Apoa5* KO mice. We performed Western blots for APOD as well, but although the data were consistent with a modest increase in *Apoa5* KO VLDL, the relevant bands were weak and required long exposures to visualize (Supplemental Fig. 3). Also recapitulated was the striking decrease of SAA and APOE in *Apoa5* KO mice versus the WT mice. These data lend confidence that two separate MS analyses, both run with consideration for multiple comparisons, accurately reflected differences in protein levels in VLDL between the two genotypes.

### Introduction of r-APOA5 partially normalizes the VLDL proteome in mouse plasma

**Fig. 7:**
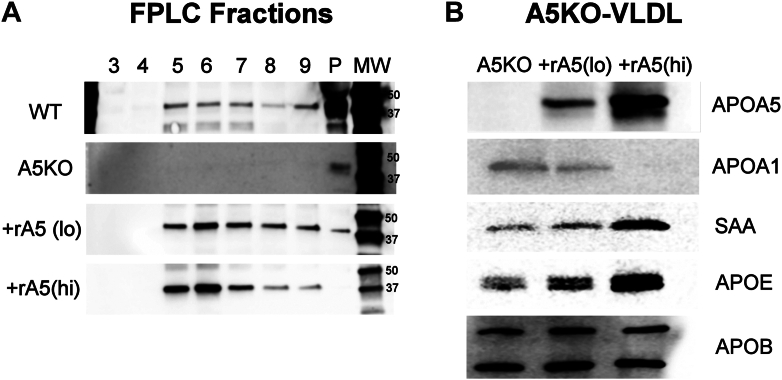
Effect of the addition of r-APOA5 to *Apoa5* KO plasma on VLDL proteome. A: r-APOA5 was added at two different doses to *Apoa5* KO plasma and incubated for 1 h at 37°C. VLDL was then fractionated by size-exclusion chromatography (traces are shown in Supplemental Fig. S5), and the levels of mouse APOA5 were probed by Western blot. B: The VLDL fractions 4–8 in A were pooled, concentrated, and probed for other apolipoproteins by Western blot (loaded at equal APOB levels). N = 3 mice per group with representative data shown from one mouse in each group.

To determine if these VLDL proteome changes were reversible, we took plasma from *Apoa5* KO mice and reintroduced r-APOA5 at two different doses that approximate the mouse injections shown in Figure 2. After 1 h at 37°C, we fractionated the plasma on a size-exclusion column capable of isolating the VLDL particles from HDL. Figure 7A shows that plasma from WT mice contains endogenous APOA5 in fractions 5–9, which is, of course, absent in *Apoa5* KO plasma. The lower two panels show the successful reintroduction of r-APOA5 with the low dose roughly mimicking WT plasma and the high dose showing a 2–3-fold enrichment of APOA5. The corresponding size-exclusion chromatograms are shown in Supplemental Fig. S4. The VLDL fractions were then pooled, concentrated, and then Western blotted for several of the key proteins shown to be altered in the MS studies (Fig. 7B, Supplemental Fig. S5). APOA1 levels in *Apoa5* KO VLDL, which were elevated with respect to WT VLDL, decreased dose-dependently upon reintroduction of APOA5. APOE and SAA1, which exhibited depressed levels in the *Apoa5* KO versus WT VLDL, increased in a dose-dependent manner with r-APOA5 introduction. These changes were noted at constant levels of APOB, indicating changes in total copy numbers of these proteins per VLDL particle.

### Restoration of mouse APOA5 normalizes the uptake of Apoa5 KO VLDL by cultured hepatocytes without affecting LPL lipolysis

**Fig. 8:**
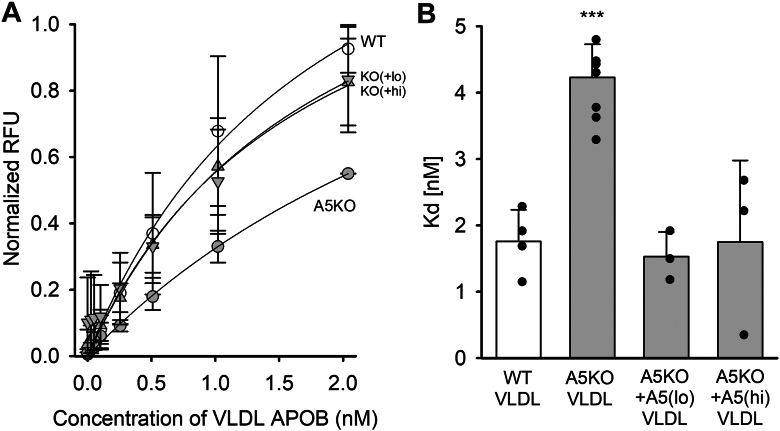
Effect of restoring r-APOA5 to *Apoa5* KO plasma on VLDL binding to cultured hepatocytes. A: WT and *Apoa5* KO plasma were incubated with DiI to label all lipoproteins. The KO DiI-plasma was separated into three groups and treated with PBS (negative control), 500 ng/ml (2×), and 2.5 μg/ml (10×) r-APOA5 for 1 h at 37°C. WT plasma (positive control) was treated with PBS. VLDL was separated from plasma by FPLC, and uptake assays were performed using cultured AML12 cells at the indicated concentrations of DiI-VLDL. The figure shown is an average of at least three experiments, each performed in triplicate (WT n = 4, A5KO n = 6, A5KO+A5 [low and high] n = 3). The values were normalized to cell protein as well as the intensity of fluorescence labeling between groups. WT VLDL (open circles), *Apoa5* KO VLDL (gray circles), *Apoa5* KO plus the low dose of r-APOA5 (down triangles), *Apoa5* KO VLDL plus high dose r-APOA5 (up triangles). B: The apparent *K*_*d*_ of binding/uptake measured as in Figure 3. All values show means, and error bars show 1 SD. ∗∗∗ shows statistically significant difference from WT condition by one-way ANOVA (Bonferroni post-test).

We next evaluated the impact of restoration of mouse APOA5 on two functional aspects of the resulting VLDL particles. Using a similar protocol as Figures 6 and 7, we incubated *Apoa5* KO plasma with increasing doses of r-APOA5 for 1 h at 37°C. We then added exogenous LPL to the plasma samples as in Figure 2. Although we measured orlistat-sensitive generation of NEFAs, we noted no APOA5-mediated increase in lipolysis (Supplemental Fig. S6). Again, this is consistent with the idea that APOA5 on its own neither directly stimulates LPL nor causes changes in VLDL proteins known to affect LPL activity (such as altering APOC2, e.g.). We next evaluated the effect of two doses of r-APOA5 on the ability of isolated VLDL particles to bind and be cleared by mouse hepatocytes. As in Figure 7, the r-APOA5 was incubated in unfractionated plasma prior to separation. Supplemental Fig. S4 shows that the addition of the recombinant protein had no major effect on TG, cholesterol, or phospholipid distribution between VLDL (fractions 3–9) or HDL (fractions 19–27). The VLDL fractions were pooled, and the particle binding to cultured hepatocytes was assayed (Fig. 8). As in Figure 3, VLDL isolated from WT mice showed robust binding, whereas those from the *Apoa5* KO were cleared significantly slower. Figure 8A shows that prior incubation with both doses of r-APOA5 sped up particle clearance, which was likely attributed to a normalization of binding capacity, as determined by *K*_*d*_ values (Fig. 8B).

## Discussion

Until recently, the low abundance of APOA5 in plasma has posed a significant challenge to understanding its role in TG metabolism. Simply put, there did not appear to be enough of the protein for the kind of direct protein-protein interactions typically associated with the regulation of TGRL hydrolysis. For instance, it is difficult to envision a scenario in which APOA5 directly binds to and activates LPL, given that APOA5 circulates at a concentration of about 6 nM (14), whereas LPL is present at approximately 93 nM in normal plasma and can reach ∼850 nM in heparinized plasma (30). LPL concentrations are likely even higher in localized environments, such as at the surface of endothelial cells, further stretching the plausibility of a direct stoichiometric interaction. Given the known higher intracellular concentration of APOA5 in hepatocytes versus plasma (31), Shu *et al.* (32) hypothesized an intracellular function—specifically, that APOA5 alters APOB lipidation without affecting its secretion rate. That idea faltered when they found APOA5 did not colocalize with APOB during VLDL assembly and secretion. However, they did observe strong localization of APOA5 to intracellular lipid droplets in hepatocytes, suggesting it might play a role in apportioning stored lipid to the lipoprotein secretion pathway. This led us to consider an alternative role for APOA5. Rather than a direct interaction between APOA5 and APOB to modulate lipid incorporation, we hypothesized that APOA5 might act intracellularly to "program" VLDL particles with specific apolipoprotein combinations known to modulate LPL activity. For instance, APOA5-driven enrichment of outgoing VLDL with APOC2 (an LPL cofactor) and/or reduction of APOC3 (an inhibitor) could help explain how APOA5 enhances LPL activity despite its minimal presence in plasma. This idea received some initial support from Pennacchio *et al.* (1), who showed limited evidence for altered APOC levels in VLDL from Apoa5 KO mice. Unfortunately, our data struck this hypothesis down. If hepatic APOA5 were responsible for programming the VLDL proteome to enhance LPL activity, then VLDL from WT mice would be expected to undergo greater lipolysis than that from Apoa5 KO mice when exposed to exogenous LPL in the absence of other plasma factors. Instead, as shown in Figure 2, the opposite was observed: VLDL from *Apoa5* KO mice exhibited increased lipolysis. Furthermore, our proteomic analysis revealed no differences in APOC1, C2, or C3 content between WT and *Apoa5* KO VLDL, when compared on a per-particle basis. In fact, with the possible exception of APOE (25), we found few established connections between any of the proteins that varied on VLDL between the two genotypes and LPL activation/inhibition. Around the same time, Chen *et al.* (14) published compelling evidence that a primary mechanism by which APOA5 enhances LPL activity is via binding to the ANGPTL3/8 complex, neutralizing its inhibitory effect on LPL activity. More recent work shows that the ANGPTL3/8 complex works by irreversibly denaturing LPL, offering a satisfying explanation of how low circulating concentrations of this complex (∼0.1 nM) can inhibit intravascular LPL, whereas low concentrations of its derepressor, APOA5, can regulate the process (33).

Nevertheless, our data clearly show that the absence of APOA5 significantly remodels circulating VLDL. *Apoa5* KO VLDL was overall depleted in total protein versus WT without changes in the ratio of PC to TG in the particles. We noticed that many of the proteins reduced in *Apoa5* KO VLDL participate in inflammatory processes (SAA1, PCYOX1, CLU, and PON1), protease function (SPINK3, CST3, and ITIH4), or immune function (C4b, C3, CLU, and H2-Q10). Recent studies (34) have revealed that lipoproteins carry complex proteomes with many protein functions going well beyond lipid transport and into inflammation, hemostasis, and the innate immune system. The reduction of these proteins, particularly the acute phase response protein SAA, in the absence of APOA5 seems counterintuitive given the expectation that persistent circulating TGRL might promote an inflammatory state. Indeed, deletion of *Apoa5* in hamsters led to hepatic steatosis even on a chow diet (35), and *Apoa5* knockdown in mice resulted in increased cytokine markers of systemic inflammation, including IL-10, IL-6, and TNF-α (36). The relationship between systemic inflammatory state and the VLDL proteome will clearly need further study.

Although the absence of APOA5 did not affect LPL regulatory proteins in VLDL, we observed notable changes in APOE, a key mediator of remnant TGRL clearance by the liver. APOE typically accumulates on VLDL particles as they shrink during systemic lipolysis, facilitating their binding to the LDL receptor (LDLr), LDLr-related protein 1, and hepatic cell surface heparan sulfate proteoglycans (HSPGs). Our MS and Western blot analyses revealed that APOE levels were markedly reduced in VLDL from *Apoa5* KO mice compared with WT. Moreover, incubating recombinant APOA5 with *Apoa5* KO plasma resulted in a redistribution of APOE to yield VLDL particles enriched in APOE. This enrichment correlated with enhanced binding and uptake by cultured hepatocytes. These interventional experiments confirm that APOA5 modulates APOE content on VLDL, influencing hepatic clearance and contributing to elevated circulating TG, alongside its effects on LPL activity through the ANGPTL3/8 pathway discussed above.

How does APOA5 encourage the appearance of APOE on VLDL? APOE is well known to accumulate on TGRL remnants as they undergo TG depletion in the circulation (37, 38) and becomes a major ligand for liver clearance of those particles via LDLr, LRP, and HSPGs (39). The reason that APOE moves to these particles is not clear, but it may relate to favorable surface curvature characteristics as the particle decreases in size. In the absence of APOA5, LPL-mediated lipid hydrolysis is impaired, and VLDL particles shrink at a slower rate and therefore may not attract as much APOE. Thus, one might argue that many of our results are simply consistent with increased ANGPTL3/8 inhibition of LPL in the *Apoa5* KO animals, which would slow VLDL processing and explain the lower levels of APOE. However, our size-exclusion chromatography analysis of VLDL and *Apoa5* KO plasma showed no major differences in particle size under the fasted conditions used here (Supplemental Fig. S1). Also, in our in vitro experiments, *Apoa5* KO plasma (nonheparinized) lacked significant lipase activity. Yet, VLDL particles in this experiment still became enriched in APOE when we added recombinant APOA5. This suggests a more direct explanation for the effect than particle size changes during lipolysis. One possibility is that APOA5 creates biophysical conditions that allow more APOE to bind to VLDL. For example, it could alter surface lipid-packing characteristics that favor APOE incorporation from other lipoproteins, such as HDL. However, this seems unlikely when one considers the sparse number of APOA5 molecules on each VLDL (about 1 per 25 particles). Our study was limited in that we could only assess what happens when relatively large concentrations of APOA5 are incubated directly in mouse plasma. In the circulation in vivo, we doubt that VLDL particles encounter high enough concentrations of APOA5 to cause the proteomic changes we saw here. However, APOA5 concentrations in the mouse liver are substantially higher than in the circulation (31), and one could easily imagine such APOA5 displacement effects happening directly at the site of VLDL synthesis in the hepatocyte. Another possibility is that APOA5 works on the surface of other lipoproteins to displace APOE, which can then migrate to VLDL. APOA5 is distributed between VLDL and HDL in normal plasma. Since APOE resides on roughly 9% of HDL particles (40), the presence of APOA5 might limit APOE’s affinity for HDL, shifting the equilibrium toward VLDL binding.

Our data also showed that VLDL from *Apoa5* KO mice was significantly enriched in A2M, a broad-spectrum protease inhibitor that, like APOE in TGRL remnants, also binds LRP (41). We considered the idea that A2M might compete with APOE for binding LRP as a potential explanation for the reduced clearance of *Apoa5* KO VLDL in our cultured hepatocytes. Although some have suggested that A2M and APOE compete for LRP binding (42), more recent studies have shown that A2M and APOE likely bind to two different sites on the receptor (43). Interestingly, A2M has also been shown to directly bind APOE (44); therefore, it is possible that some APOE on *Apoa5* KO VLDL particles is complexed with A2M and perhaps less available for interactions with LRP. Finally, it is worth noting that residues toward the C terminus of SAA have been implicated in binding to HSPGs (45) and receptors LOX 1, SR-B1, and CD36 (26, 46, 47), all of which are present on hepatocytes. Though the role of SAA on VLDL clearance has not been examined, it is possible that SAA may contribute to WT VLDL interactions with hepatocytes and possibly their clearance from the system. Since their expression is reduced in the VLDL of *Apoa5* KO, this could be another possible mechanism for TG buildup in *Apoa5* KO mice.

This study offers the most comprehensive proteomic comparison to date of VLDL particles from *Apoa5* KO and WT mice. While the Konrad and Young laboratories (14, 15, 16) have beautifully described the role of APOA5 in guiding LPL location and activity through its interaction with the ANGPTL3/8 complex, our findings reveal another functional mode: APOA5 promotes the accumulation of APOE, a key ligand for hepatic clearance of remnant VLDL particles, and possibly other similar ligands. Exploitation of APOA5 as a potential TG-lowering therapeutic faces numerous challenges because protein levels would likely need to be boosted only in the circulation to prevent unintended intracellular effects on liver lipid droplet metabolism. A more straightforward strategy for promoting TG hydrolysis in circulation involves targeting two well-characterized inhibitors of LPL activity, APOC3 and ANGPTL3, both of which are the focus of ongoing clinical trials (48). However, the long-term impact of sustained lipolysis and increased fatty acid delivery to peripheral tissues remains unclear. An optimal therapeutic approach may need to not only enhance lipolysis but also accelerate the hepatic clearance of remnant lipoproteins. A deeper understanding of APOA5’s effects on APOE movement may point the way toward achieving this balance.

## Data availability

The MS proteomics data obtained from both sites A and B have been deposited to the ProteomeXchange Consortium via the PRIDE (27) partner repository with the dataset identifier PXD065274.

## Supplemental data

This article contains supplemental data.

## Conflict of interest

The authors declare that they have no conflicts of interest with the contents of this article.
