# Supplementary material for: Apolipoprotein A5 reduces clearance of VLDL by altering apolipoprotein E content
Source: J Lipid Res. 2025 Oct 1;66(11):100917. doi: 10.1016/j.jlr.2025.100917 (PMC12615743; doi:10.1016/j.jlr.2025.100917)
Supplement: Supplementary Figures and Tables [file mmc1.docx]

**ONLINE DATA SUPPLEMENT**

**Apolipoprotein A5 Reduces Clearance of Very Low-density Lipoprotein by Altering Apolipoprotein E Content**

Pheruza Tarapore^1^, Debi Swertfeger^2^, Jamie Morris^1^, Yi He^3^, Snigdha Sarkar^4^, John T. Melchior^1,4^, Amy S. Shah^2^, Min Liu^1^ and W. Sean Davidson^1*^

^1^ Department of Pathology and Laboratory Medicine, University of Cincinnati, Cincinnati, OH, USA

^2^ Department of Pediatrics, Cincinnati Children’s Hospital Medical Center and the University of Cincinnati, Cincinnati, OH, USA

^3^ Department of Medicine, University of Washington School of Medicine, Seattle, WA, USA

^4^ Biological Sciences Division, Earth and Biological Sciences Directorate, Pacific Northwest National Laboratory, Richland, WA, USA

* To whom correspondence should be addressed: W. Sean Davidson: Department of Pathology and Laboratory Medicine, University of Cincinnati, 2120 Galbraith Rd., Cincinnati, Ohio 45237-0507 USA, Tel.: (513) 558-3707; Fax: (513) 558-1312; E-mail: Davidswm@ucmail.uc.edu

Supplemental Figure 1


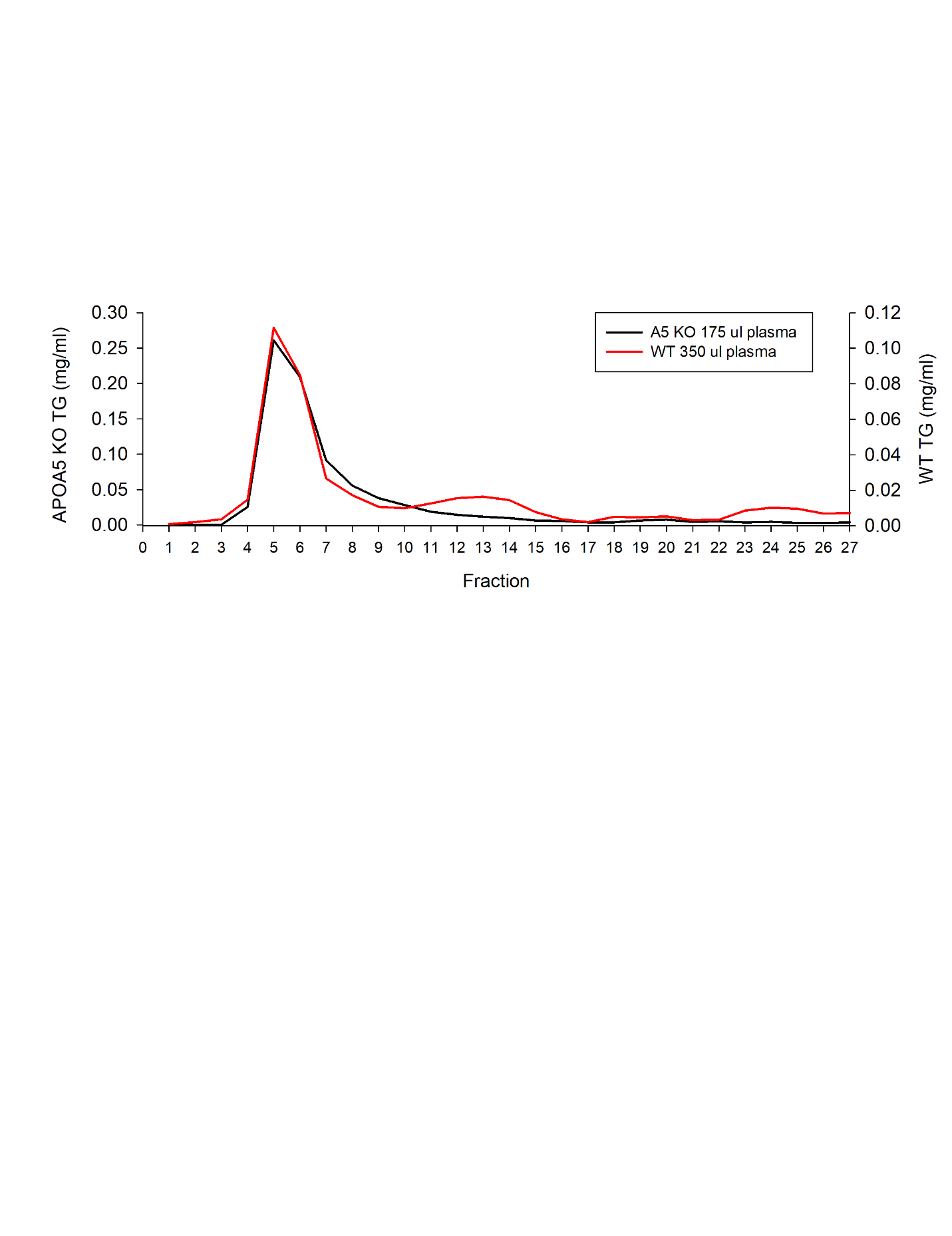
**Figure S1. Comparison of VLDL particle size in baseline WT and *Apoa5* KO mice.** Mouse plasma was run on a Superose 6 gel filtration column set up on an FPLC and 0.5 ml fractions were collected across the separation range. Triglyceride was monitored in each fraction using enzymatic kits (see *Methods*). These traces come from a single animal each, but the experiment was performed in three mice for each group. Because *Apoa5* KO mice exhibit significantly higher fasting levels of TG than WT mice (see **Fig. 1b** in the paper), 350 µl of WT plasma was injected onto the column vs. 175 µl for *Apoa5* KO plasma. Even so, the data needed two different Y-axes. We normalized the peak size of the VLDL peak so that a direct comparison of initial particle size could be made.

Supplemental Figure 2


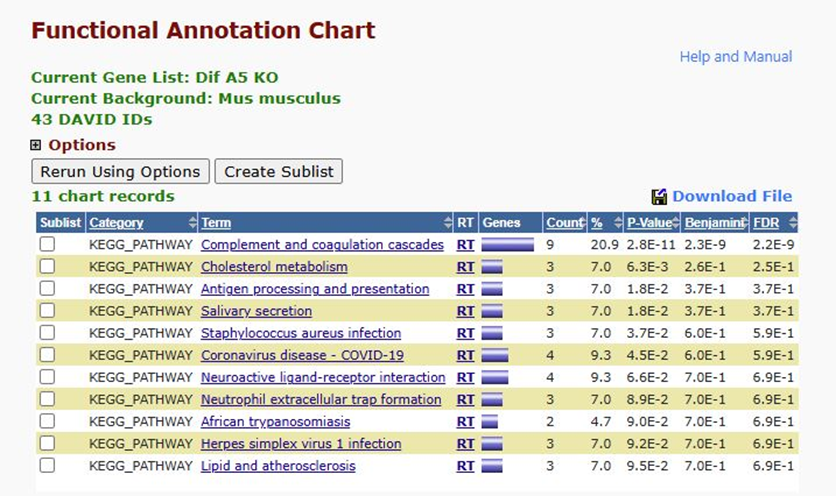


**Figure S2. Gene ontology analysis of proteins that differed in abundance between WT and *Apoa5* KO VLDL**. The analysis was performed on the DAVID server using KEGG pathway categories.

Supplemental Figure 3


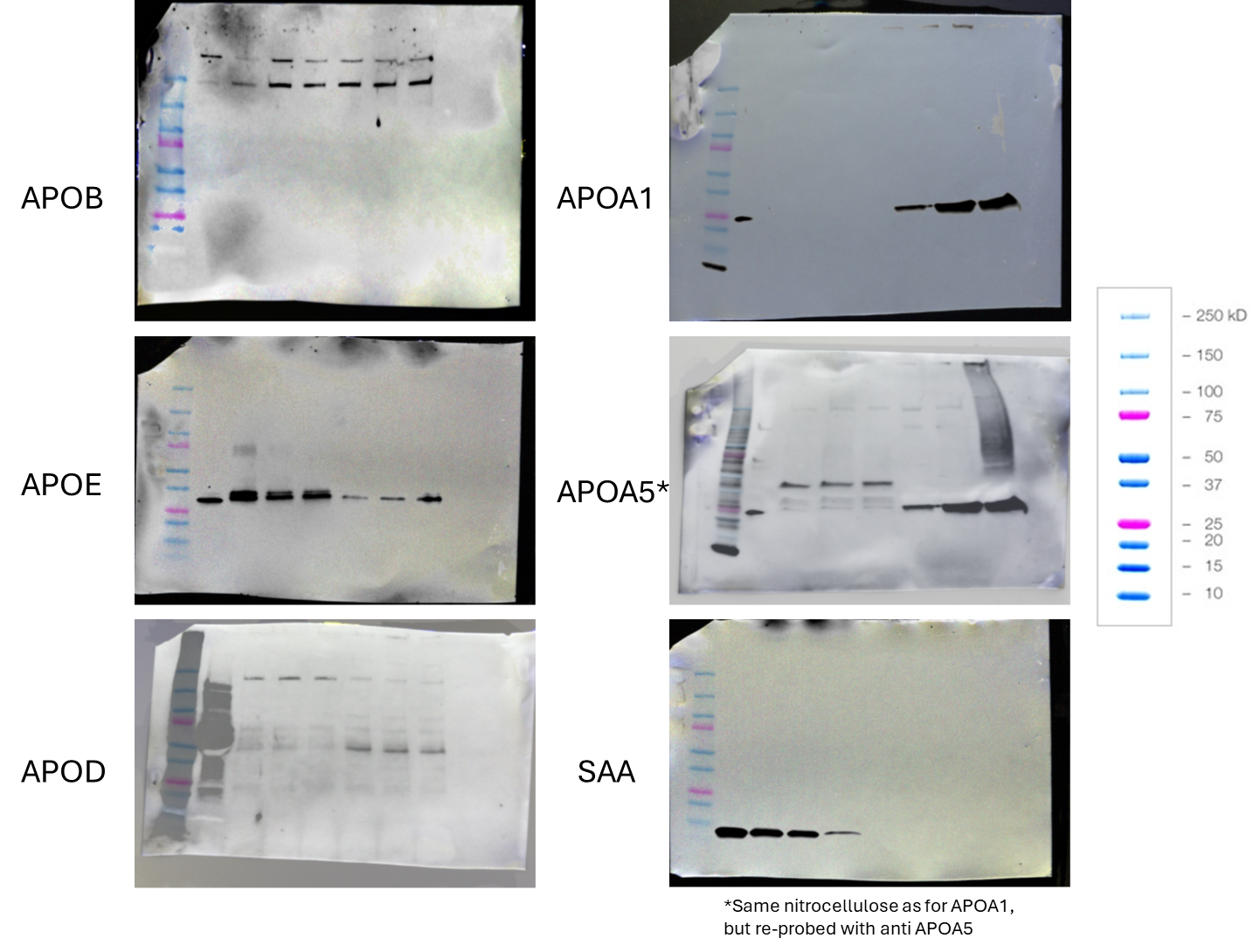


**Figure S3. Uncropped western blots for images shown in Fig. 6.** All western blots are set up identically with: lane 1 containing BioRad Precision Plus^TM^ colored protein standards (see MW key on the right); lane 1 containing diluted WT mouse plasma to show size of endogenous protein, 2,3,4 containing WT animals 1, 2 and 3; lanes 4,5,6 containing *Apoa5* KO animals 1, 2, and 3.

Supplemental Figure 4


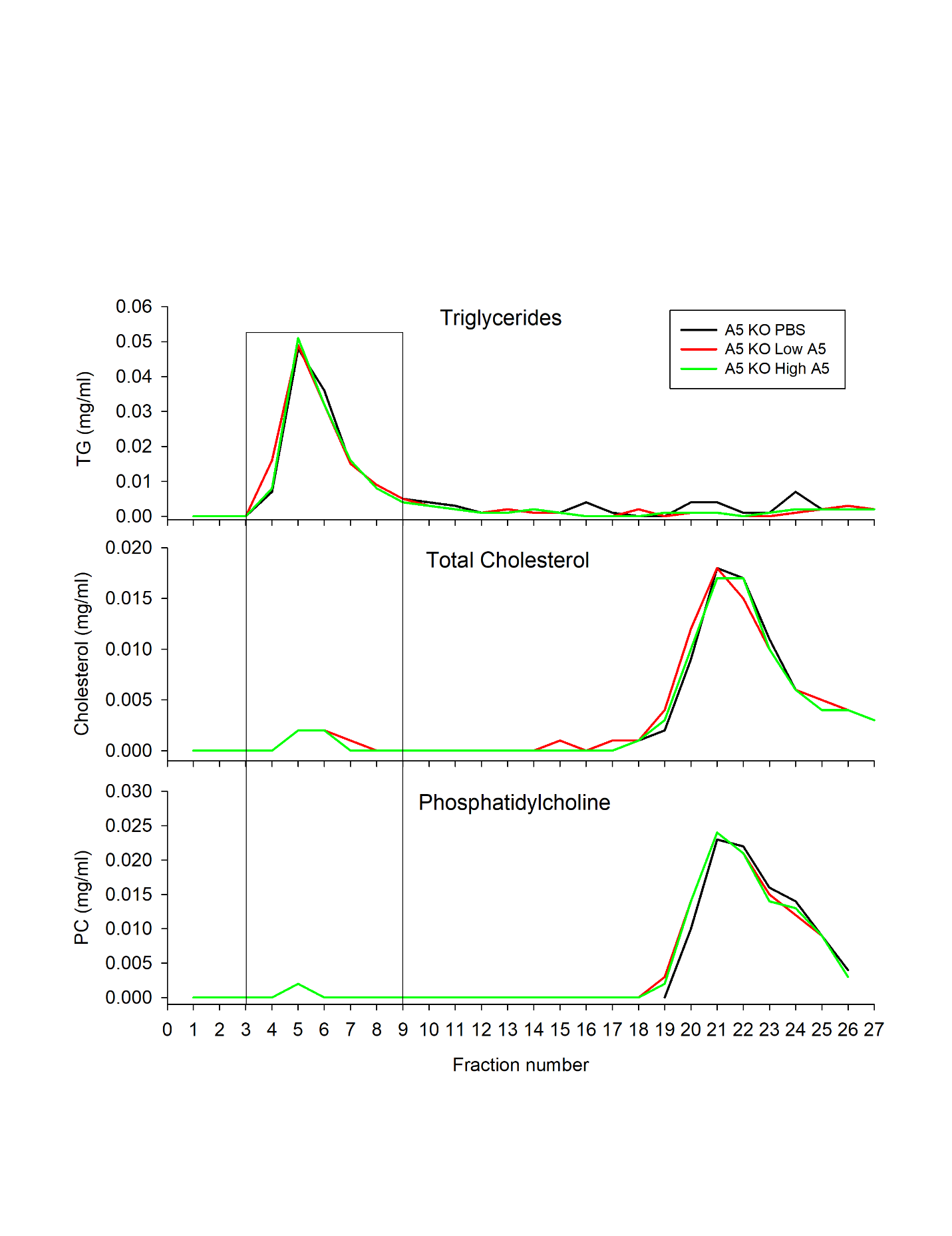


**Figure S4. Size exclusion chromatography of mouse plasma with added recombinant APOA5.** Mouse plasma was run on a tandem Superose 200 gel filtration column setup on an FPLC and 0.5 ml fractions were collected across the separation range. Triglyceride (top), total cholesterol, and choline-containing phospholipids were monitored in each fraction using enzymatic kits (see *Methods*). These traces come from a single animal each, but the experiment was performed in triplicate with similar traces for each. The fractions that were western blotted for APOA5 in **Fig. 7a** are shown in the boxed area.

Supplemental Figure 5


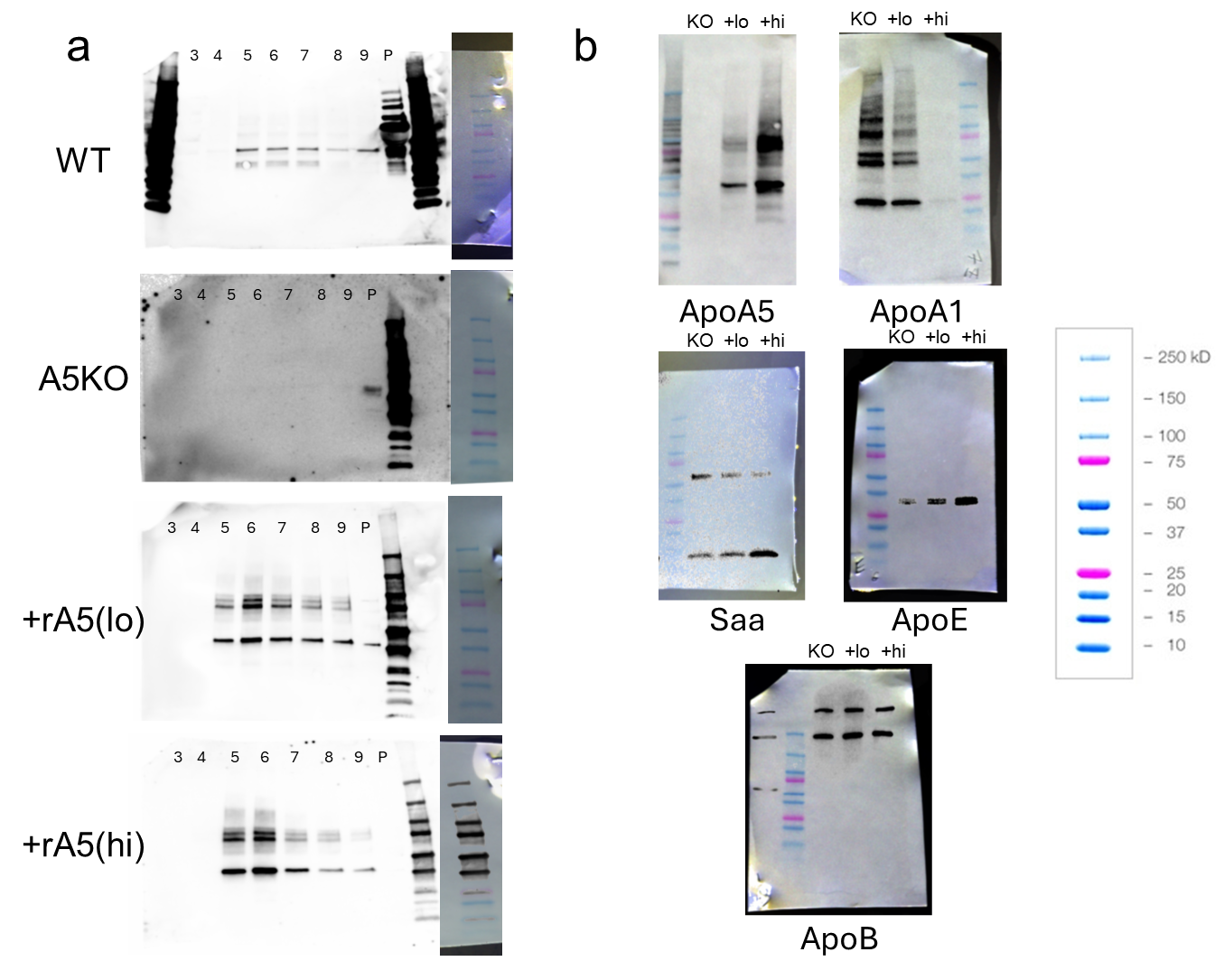


**Figure S5. Uncropped western blots for images shown in Fig. 7.** (**a**) All western blots are set up similarly with the lanes marked with the fraction numbers as in Fig. 7 and corresponding to the FPLC traces in **Fig. S4**. Lane P shows diluted WT mouse plasma to show size of endogenous protein. Rightmost lane contains BioRad Precision Plus^TM^ colored protein standards (see MW key on the right). Placed next to the black and white blots is a color version of the blot with colored standards apparent. (**b**) All western blots are set up similarly with MW standards on the left (or right in the case of APOA1). The lane labeled KO refers to *Apoa5* KO mice with no additions to their plasma, +lo indicates the low dose of recombinant *Apoa5* added, and +hi indicates the high dose of recombinant APOA5 added.

Supplemental Figure 6


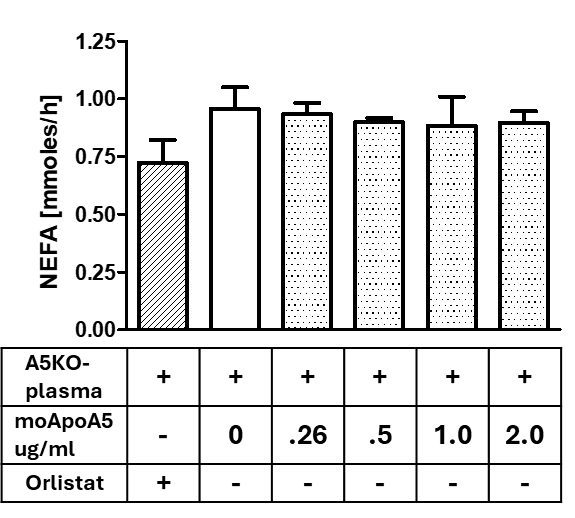


**Figure S6. Effect of re-introduction of r-APOA5 on triglyceride lipolysis in unfractionated plasma of *Apoa5* KO mice.** **(a)** Recombinant mouse APOA5 was incubated with plasma for 1 h at 37 º. Exogenous LPL was added and generation of free fatty acids was measured as in **Fig. 2**. The data is from two experiments performed in triplicate. The values (mean ± 1 SD) were normalized to cell protein as well as the intensity of fluorescence labeling between the groups.
